# Direct and Indirect Effects of a Glyphosate-Based Herbicide on Spodoptera frugiperda Multiple Nucleopolyhedrovirus (*Baculoviridae*) on Diet, Maize Plants and Soil

**DOI:** 10.3390/insects17010073

**Published:** 2026-01-08

**Authors:** Juan S. Gómez-Díaz, Arely Y. Cubas, Mara J. Arias-Robledo, Trevor Williams

**Affiliations:** Instituto de Ecología AC, Xalapa 91073, Veracruz, Mexico; juan.gomez@inecol.mx (J.S.G.-D.); arelyyared@hotmail.com (A.Y.C.); arias.mara@outlook.com (M.J.A.-R.)

**Keywords:** *Alphabaculovirus*, fall armyworm, biological effects, infectivity, larval susceptibility, soil persistence, virus-herbicide interaction

## Abstract

Glyphosate is a broad-spectrum, systemic herbicide that has attracted concern over its non-target effects, environmental persistence, and the presence of residues in food. The fall armyworm, *Spodoptera frugiperda* (Lepidoptera: Noctuidae), is a major invasive pest of maize that can be controlled by application of its homologous nucleopolyhedrovirus (SfMNPV), an occluded virus in the family *Baculoviridae*. We examined the effects of a glyphosate-based herbicide on *S. frugiperda* growth and survival and on virus occlusion bodies (OBs) exposed to product label-recommended concentrations of the herbicide. Larval growth, time to pupation, pupal weight, duration of the pupal stage and sex ratio were not affected by exposure to the herbicide (1% *v*/*v* solution) applied to the surface of semi-synthetic diet. Exposure to 1–2% herbicide solution had no effect on the median lethal concentration (LC_50_) of OBs, the susceptibility of second instar larvae to virus infection, or the production of OBs in virus-killed larvae. Virus acquisition did not vary significantly when larvae fed on virus-sprayed maize plants at 1 and 6 days after they had been treated with herbicide, compared to healthy plants. Finally, the presence of 2% herbicide solution did not influence the persistence of OBs in non-sterilized soil samples over a 6-week greenhouse experiment. Although the laboratory and greenhouse experiments indicated that the glyphosate-based herbicide tested was unlikely to influence the transmission or persistence of SfMNPV OBs, future studies should verify these findings across a range of field conditions, soil types and different herbicide formulations.

## Simple Summary

Glyphosate is a widely used herbicide that has attracted concern over its potential effects on a diversity of animals, including aquatic organisms and beneficial insects, its persistence in the environment, and the presence of herbicide residues in human food. The fall armyworm, *Spodoptera frugiperda* is a major pest of maize in many parts of the world. The larvae of this pest can be controlled by applications of its nucleopolyhedrovirus. In a series of experiments, we observed that the growth, pupation, and sex ratio of the fall armyworm were not affected by exposure to recommended rates of the herbicide. Similarly, the presence of herbicide had no effect on the stability, infectivity or soil persistence of the virus, or the acquisition of infection from virus-sprayed herbicide-treated maize plants. Although these laboratory and greenhouse findings suggest that the glyphosate-based herbicide we tested is unlikely to influence the efficacy of this virus as a biological insecticide, these results should be verified in field studies across a range of soil types and different herbicide formulations.

## 1. Introduction

Lepidopteran nucleopolyhedroviruses (*Alphabaculovirus*, *Baculoviridae*) are virulent pathogens that form the basis for effective biological insecticides for the control of lepidopteran pests of field and greenhouse crops and in sensitive forest ecosystems [1].

Larvae become infected after consuming foliage contaminated with viral occlusion bodies (OBs). The OBs dissolve in the alkaline conditions of the insect midgut, releasing occlusion-derived virions (ODVs) that infect midgut columnar cells (primary infection) from which the virus spreads to other tissues in the form of budded virions that are responsible for the systemic secondary infection. Later in infection, progeny OBs accumulate in the cell nucleus and are released following the death and liquefaction of the insect by virally-encoded enzymes [2]. The OBs can persist on plant foliage or in the soil until they are consumed by susceptible host larvae to initiate the following cycle of transmission [3].

The fall armyworm, *Spodoptera frugiperda* (Lepidoptera: Noctuidae), is a major polyphagous pest that has recently spread across much of the world, resulting in major losses in maize and a range of other crops [4]. The use of the Spodoptera frugiperda multiple nucleopolyhedrovirus (SfMNPV; species name, *Alphabaculovirus spofrugiperdae*) as the basis for biological insecticides against this pest is of rapidly growing interest in the Americas (native range) and across its newly established range [5,6,7,8].

Although studies on the interaction of nucleopolyhedroviruses and other insect pathogens have long attracted the interest of insect pathologists and biocontrol workers [9,10], there is a limited understanding of the interaction of nucleopolyhedroviruses with agrochemicals [11,12,13]. Studies that have examined such interactions are mainly focused on insecticides [14,15,16], whereas interactions with fungicides and herbicides are noticeably more infrequent despite the widespread use of these products in modern agriculture [13,17].

Glyphosate (N-phosphonomethyl glycine) is a broad-spectrum, systemic herbicide that acts by inhibiting the shikimate pathway for aromatic amino acid synthesis in plants. Intensive use of this compound and its increasing presence in the environment have triggered a series of concerns regarding its toxicity to non-target organisms [18], including beneficial insects [19,20]. Glyphosate has also been linked to potential human health risks, including endocrine disruption, cancer and neurological issues [21], although from a regulatory perspective this compound is currently considered to be safe for use in many countries, including the USA and the European Union [22]. In Mexico, the President of Mexico decreed a complete ban on the use of glyphosate beginning in 2024, although the federal government has temporarily suspended the ban until effective alternative products can be identified [23].

Glyphosate is widely used in maize production as a pre-sowing burndown treatment to eliminate weeds, particularly in low- or no-tillage systems [24], and is also used as a post-emergence treatment in countries that allow the use of genetically-modified glyphosate-resistant varieties of maize [25,26]. As a post-emergence treatment, weed control is most important during the ~6-week period after sowing when crop yields are most affected by competition from weeds [27,28]. This is the period when maize is most susceptible to the attack of *S. frugiperda* larvae, which can act as cutworms on seedings and consume the developing leaves in whorl-stage maize [29]. As SfMNPV-based insecticides are also applied during this period to control *S. frugiperda* larvae, it is highly likely that OBs sprayed onto plants, or released from virus-killed larvae, would experience the presence of glyphosate herbicide residues on plant surfaces or in the soil.

Herbicides could interact with insect pathogenic viruses in multiple ways, both directly and indirectly. Direct effects could relate to effects on the physical stability or functional integrity of the OB or its constituent ODVs, protein matrix or the exterior polyhedral envelope [30]. Indirect effects include those related to the development, feeding behavior or intrinsic susceptibility of the host insect, or by affecting the persistence of viral OBs in the environment.

In the present study we aimed to evaluate the effects of a glyphosate-based herbicide on the growth of *S. frugiperda* larvae, the pathogenicity of SfMNPV OBs, and on the survival of insects that consumed mixtures of herbicide and OBs on semi-synthetic diet, effects on the acquisition of virus infection from glyphosate-treated plants and the effects on short-term persistence of SfMNPV OBs in soil. To our knowledge, this is the first study to examine glyphosate herbicide effects on OB stability, nucleopolyhedrovirus acquisition from contaminated plants and soil OB persistence.

## 2. Materials and Methods

### 2.1. Insects, Virus and Herbicide

Larvae of the fall armyworm, *S. frugiperda* were obtained from a laboratory colony maintained in the Instituto de Ecología AC. This colony was started using larvae collected from maize fields close to Zempoala, Veracruz, Mexico (19°26′14″ N, 96°22′40″ W). The colony was maintained at 26 ± 1 °C, 70 ± 10% RH, 14:8 h L:D photoperiod. Larvae were reared on a semi-synthetic diet based on soybean flour, brewer’s yeast, wheat germ, vitamins and salts (Supplementary Table S1). PCR analyses indicated that the colony was free from SfMNPV infection [31]. All the experiments described in the following sections were performed using semi-synthetic diet that did not contain formalin.

A Nicaraguan isolate of SfMNPV (SfMNPV-NIC) [32], was produced by inoculating fourth instars of *S. frugiperda* using the droplet feeding technique. Fourth instars were used due to the large quantities of OBs that can be produced in late instar larvae. SfMNPV-NIC is a genotypically diverse natural isolate that comprises at least nine genotypic variants [33]. Inoculated larvae were incubated individually with diet in darkness at 27 ± 1 °C until death. Virus-killed larvae were macerated in ultrapure water (GenPure xCAD Plus, Barnstead Water Purification Systems, Thermo Fisher Scientific, Waltham, MA, USA) and filtered through an 80 μm steel mesh. The resulting suspension was placed in 1.5 mL microcentrifuge tubes and centrifuged at 400× *g* for 6 min to sediment insect debris. The supernatant was then passed through a layer of 40% (*v*/*v*) glycerol by centrifugation at 5900× *g* for 15 min. OBs were then washed once, resuspended in 500 μL of ultrapure water and samples were counted three times in an improved Neubauer chamber.

Glyphosate was obtained as an aqueous formulation of the commercial herbicide, Takle 360 AS (Sifatec, Tlalnepantla, Mexico) containing 360 g i.a./L of glyphosate isopropylamine as the active ingredient (a.i.). Laboratory experiments were performed using this product at concentrations of 10 mL/L (1% *v*/*v*, 3.6 g a.i./L or 16 mM) or 20 mL/L (2% *v*/*v*, 7.2 g a.i./L or 32 mM), equivalent to the product label recommendation of 2 L/ha of product, assuming a total application volume of 200 or 100 L/ha of water, respectively. A soil persistence experiment was performed using the maximum label recommended rate of 50 mL/L (2.5% *v*/*v* solution, 9.0 g a.i./L or 40 mM), equivalent to 5 L herbicide/ha applied in 200 L of water. The pH of the experimental preparations was pH 5.84 (1% solution) and pH 5.78 (2% solution). These concentrations were based on the standard application rates to which viral OBs are likely to be exposed following product label recommendations. Two adjuvants are mentioned on the product label (solubilizer and surfactant), but the identity and the concentration of these substances in the product formulation remain unknown.

### 2.2. Effects of Herbicide on Insect Growth and Survival

To assess the effect of herbicide on the growth and survival of *S. frugiperda* larvae, a diet surface contamination study was performed. For this, the wells of a 12-well tissue culture plate (Costar 3537, Corning Inc., Glendale, AZ, USA) were filled to a depth of ~5 mm with semi-synthetic diet. Half the plates were treated by applying 7.6 µL (equivalent to 2 µL/cm^2^) of 1% herbicide solution to the diet surface (the equivalent of a 200 L application over an area of 1 ha), whereas the remaining plates were treated with an identical volume of ultrapure water. In both cases, the liquid was spread evenly over the diet surface using a fine paintbrush and allowed to dry for 1 h. Fourth instar larvae were weighed individually to a precision of ±0.1 mg (Explorer Analytical Balance, Ohaus Corp., Parsippany, NJ, USA) and then placed in each well of the tissue culture plate, covered with a paper towel and sealed with a lid that was perforated for ventilation. Insects were incubated in darkness at 27 ± 1 °C, checked for mortality and individually weighed at intervals of 24 h. At 48 h after pupation, the insects were sexed, weighed, placed individually in the wells of a clean tissue culture plate and monitored daily for adult eclosion. The insects were incubated at 27 °C rather than 26 °C (insectary temperature) as the laboratory incubators (Thermo Fisher Scientific Inc, Waltham, MA, USA) maintained stable temperature conditions at this setting. The experiment was performed using six groups (6 replicates) of 12 insects for the treatment and control (total = 144 insects).

### 2.3. Direct Effects of Herbicide on OB Activity

To examine the direct effects of exposure to glyphosate herbicide on OBs in suspension, a 1 mL volume of 1 × 10^8^ OBs/mL was incubated with 20 µL of herbicide (equivalent to a 2% *v*/*v* herbicide solution) at 27 ± 1 °C for 24 h in darkness. An identical OB suspension was incubated without herbicide. The suspensions were then centrifuged at 1500× *g* for 10 min to pellet OBs and the supernatant was removed and discarded. OBs were resuspended in 1 mL ultrapure water and diluted to produce concentrations of 1 × 10^3^, 3 × 10^4^, 1 × 10^5^, 1 × 10^6^ and 1 × 10^7^ OBs/mL in 10% sucrose solution with 0.05% Fluorella blue food coloring. Groups of 24 early second instars that had been starved overnight were then allowed to drink the suspensions. Insects that consumed the suspension within a 15 min period were individually placed in the wells of a 24-well tissue culture plate (Costar 3738, Corning Inc., Glendale, AZ, USA) with a piece of semi-synthetic diet and incubated in darkness at 27 ± 1 °C and checked daily for virus-induced mortality. Virus deaths were confirmed by microscopic observation of Giemsa-stained smears of insect tissues. Control larvae were treated identically but did not consume OBs. Second instar larvae were used for this experiment because they tend to have higher susceptibility and lower variation in their mortality response to OB inoculum compared to later instars [34]. The experiment was replicated on three occasions.

### 2.4. Effects of Herbicide on Virus-Induced Mortality and OB Production

A diet surface contamination bioassay was performed as described in Section 2.2, except that diet in the wells of tissue culture plates was subjected to one of the following four treatments: (i) OB suspension 1 × 10^7^ OBs/mL, (ii) OB suspension (1 × 10^7^ OBs/mL) in 1% herbicide solution, (iii) 1% herbicide solution, (iv) water control. All treatments were applied in a volume of 7.6 µL (equivalent to 2 µL/cm^2^) and spread over the diet surface as described in Section 2.2. Individual second instar *S. frugiperda* larvae were placed in each well. Plates were incubated at 27 ± 1 °C and checked at 12 h intervals for virus-induced mortality (confirmed by observation of Giemsa-stained smears) and non-specific mortality. Virus-killed larvae were collected within each replicate, homogenized in 1 mL ultrapure water, filtered through an 80 µm mesh and OBs were counted in triplicate using a Neubauer chamber to determine OB production within each replicate. The experiment was performed using five groups (5 replicates) of 12 insects per treatment.

### 2.5. Effects of Herbicide on Virus Acquisition on Treated Plants

**Figure 1:**
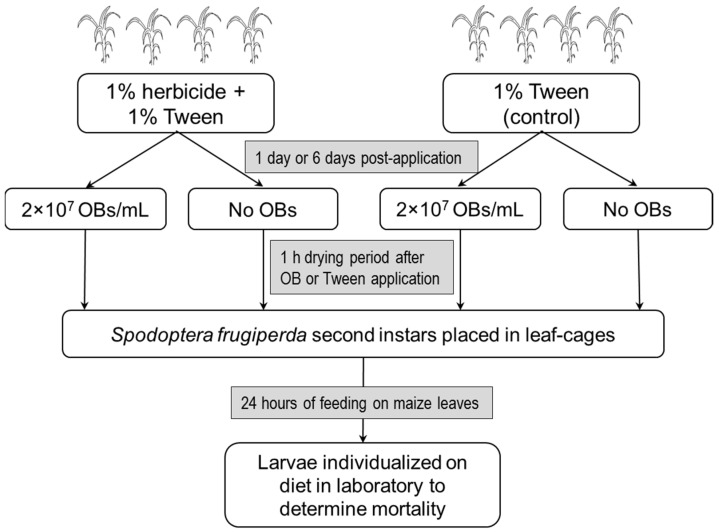
Schematic showing the sequence of experimental steps in a greenhouse experiment on virus acquisition by *Spodoptera frugiperda* second instars that fed on plants treated with glyphosate herbicide at 1 day or 6 days prior to the application of OB suspension.

Maize plants (*Zea mays*) hybrid variety H-318 (Codegram, Tenencia, Morelos, Mexico) were individually grown in soil in 250 mL polystyrene cups in an experimental greenhouse. When plants had reached a height of 35 cm with 6 true leaves, they were assigned to one of two treatment groups (i) 1% herbicide treatment or (ii) water control. Both treatments were applied in a laboratory fume extraction hood using a hand-held atomizer until run-off. Both treatments also included 1% Tween 80 as a wetting agent. Plants were placed in the greenhouse for 24 h, after which OB suspension (2 × 10^7^ OBs/mL in 1% Tween 80) was applied to run-off on half of the plants of the herbicide treatment and the control using a hand-held atomizer. The remaining plants were treated with 1% Tween 80 solution alone (Figure 1). Plants were allowed to dry for 1 h, and then leaf cages were applied to individual leaves of the maize plant (6 cages per plant). Leaf-cages were constructed from the base and foam-covered lid of a plastic Petri dish (9 cm diameter) as described previously [35]. A pair of second instar larvae was introduced to each cage and allowed to feed on the maize leaf for 24 h at 23 ± 1 °C. The cage was held in place over the leaf using an elastic band to prevent the escape of larvae. After 24 h the larvae were individualized in the wells of a 12-well plate with a piece of diet and were incubated at 27 ± 1 °C and checked daily for virus-induced mortality, which was confirmed by microscopic observation of OBs. The treated maize plants were discarded.

The procedure was repeated for plants that had been treated with herbicide 6 days previously (Figure 1). Preliminary tests indicated that herbicide-treated plants (35 cm height) began to show the first signs of glyphosate poisoning at 6 days post-treatment, with the initial signs of yellowing at the edges of some leaves, whereas at 9–12 days post-treatment, most of the leaves had wilted and died.

A total of 15 plants were subjected to each treatment combination. Each group of 12 larvae that fed on the plant was considered as one replicate.

### 2.6. Effects of Herbicide on Virus Persistence in Soil

The effect of herbicide residues on the persistence of SfMNPV OBs in soil was examined under laboratory conditions. An air-dried clay soil was sieved through a 1 mm plastic mesh and samples of 10 g were placed into opaque plastic cups (250 mL capacity) to a depth of ~1 cm. The soil had previous been characterized in terms of its morphological and physico-chemical properties [36] (Table S2). Each soil sample was moistened with 6 mL of deionized water or 6 mL of 2% (20 mL/L) herbicide solution. Soil was subjected to one of four treatments: (i) viral OBs + herbicide, (ii) viral OBs alone, (iii) herbicide control, (iv) water control. Virus treatments were applied as 5 × 10^8^ OBs in a volume of 200 µL OB suspension, whereas the treatments without OBs received 200 µL of water. Each soil sample was then mixed thoroughly with a disposable wooden spatula, and the cup was sealed with a polythene cap and weighed to a precision of ± 0.1 g. For each replicate, three cups of each treatment were prepared simultaneously: one for immediate bioassay, one for bioassay at 3 weeks and another for bioassay at 6 weeks. Cups were placed on a bench in an experimental greenhouse outside the laboratory in October 2025. A datalogger (Onset UX100-003, Hobo Data Loggers, Bourne, MA, USA) recorded temperature and humidity conditions on the bench of the greenhouse. Visible light readings were taken at intervals of 30 min between 07.00 and 18.00 on 10 days using a photometer (YK-10 LX, LT Lutron, Taipei, Taiwan). Readings were averaged over four random points measured in the vicinity of the soil samples. Each cup was weighed at weekly intervals and, if necessary, deionized water was added to maintain the soil moisture at its original value.

Soil bioassays were performed by mixing each soil sample with 90 g semi-synthetic diet using a disposable wooden spatula as described previously [37]. Small quantities of each mixture were placed in the wells of a 24-well tissue culture plate and a second instar *S. frugiperda* larva was placed in each well and allowed to feed on the mixture for 48 h at 27 ± 1 °C in darkness. Larvae were then transferred to 24-well plates with a piece of clean diet and monitored daily for virus induced mortality. When necessary, Giemsa-stained smears of larval tissues were examined under a phase-contrast microscope for the presence of abundant OBs to confirm polyhedrosis disease.

Soil bioassays were performed immediately (time point 0), 3 weeks and 6 weeks after preparing the experimental treatments. The entire experiment was replicated on four consecutive days.

### 2.7. Statistical Analysis

Changes in larval weight over time were analyzed by repeated measures analysis of variance with the Greenhouse-Geisser sphericity correction. Percentage survival to pupation was analyzed by fitting a generalized linear model (GLM) with a binomial error distribution specified. The remaining insect growth variables (time to pupation, pupal weight, duration of pupal stage and sex ratio) and larval weight at death and OB production were compared by *t*-test. In all cases, the normality and homoscedasticity of data were checked by the Shapiro–Wilk test and Levene’s test, respectively. OB activity in insect bioassays was determined by logit regression. Larval survival was subjected to Kaplan–Meier survival analysis and compared by log-rank test. The acquisition of infection in the greenhouse experiment was analyzed by paired *t*-test as maize plants treated with herbicide were set up with the corresponding virus alone treatment and controls over time. All analyses were performed using the R-based software Jamovi v.2.6 [38].

## 3. Results

### 3.1. Effects of Herbicide on Insect Growth and Survival

**Figure 2:**
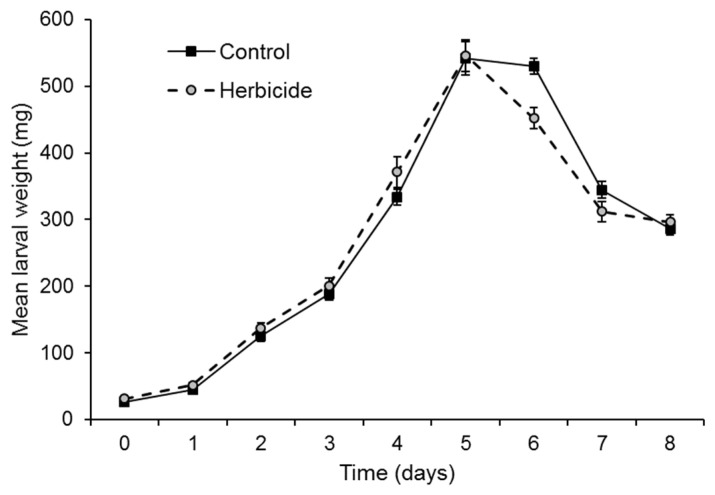
Mean weight (mg) of larvae on semi-synthetic diet surface contaminated with 1% glyphosate herbicide or water (control). Vertical bars indicate SE.

Larval weight increased markedly over time and decreased on days 6–8 as larvae prepared to pupate (*F* = 397.6; d.f. = 1.51, 15.10; *p* < 0.001), but did not differ significantly between herbicide-treated and control insects (*F* = 0.114; d.f. = 1, 10; *p* = 0.742) (Figure 2). A few insects (10 out of a total of 144 insects) that pupated after 9 days were not included in the analysis after that time.

Herbicide treatment had no significant effect on larval survival to pupation, which was high in both the treatment and control (84.7–88.9%) (GLM *χ*^2^ = 0.548; d.f. = 1; *p* = 0.459) (Table 1). In fact, compared to the control, herbicide treatment had no significant effect on any of the response variables, including the interval between the start of the experiment and observed pupation (*t* = 1.388; d.f. = 10; *p* = 0.195), the weight of pupae (both sexes) (*t* = 1.032; d.f. = 10; *p* = 0.326), the interval between pupation and adult emergence (*t* = 0.464; d.f. = 10; *p* = 0.652), and the overall sex ratio (*t* = 0.607; d.f. = 10; *p* = 0.557) (Table 1).

### 3.2. Direct Effects of Herbicide on OB Activity

Exposure to 2% herbicide solution for 24 h did not affect the OB concentration—mortality response of *S. frugiperda* second instars. Virus-induced mortality increased significantly with OB concentration (*χ*^2^ = 340.6; d.f. = 1; *p* < 0.001), but did not differ significantly between herbicide-treated and control OBs (*χ*^2^ = 0.081; d.f. = 1; *p* = 0.776). The median lethal concentration (LC_50_) values were nearly identical at 1.1 × 10^5^ OB/mL (95% CI: 8.0 × 10^4^–1.5 × 10^5^) for the untreated OBs and 1.2 × 10^5^ OB/mL (95% CI: 8.6 × 10^4^–1.7 × 10^5^) for the herbicide-treated OBs. A test for parallelism of the regression slopes (treatment × OB concentration interaction) was non-significant (*χ*^2^ = 0.142; d.f. = 1; *p* = 0.706), so that the regressions could be unified into a single regression with a consensus slope (±SE) of 0.858 ± 0.064 and a consensus intercept of −10.010 ± 0.740. There was no evidence of overdispersion in the data. None of the control larvae died from virus disease or non-specific causes.

### 3.3. Effects of Herbicide on Virus-Induced Mortality and OB Production

**Figure 3:**
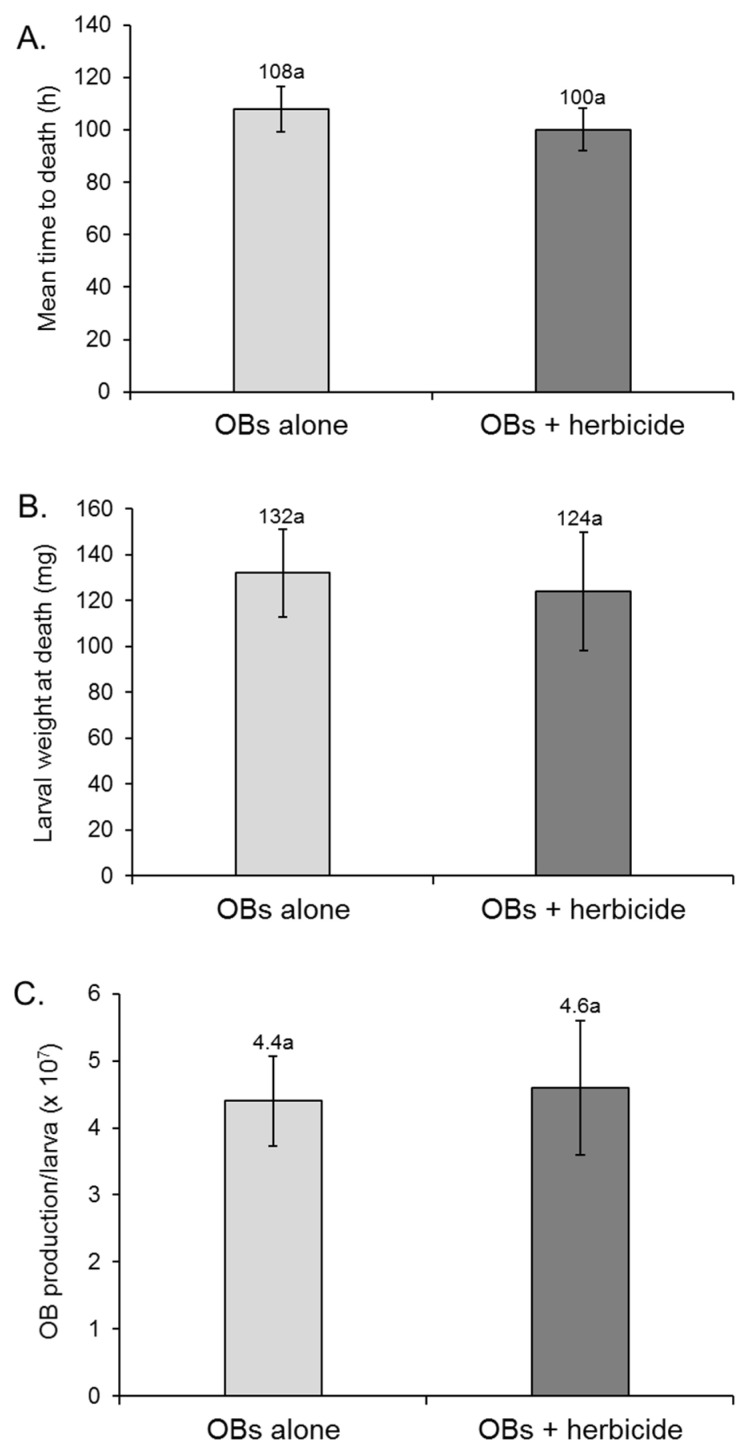
Influence of SfMNPV OB suspension applied to diet surface (OBs alone) or mixed with 1% glyphosate herbicide solution on the (**A**) mean time to death, (**B**) mean larval weight at death and (**C**) OB production/larva in *Spodoptera frugiperda* larvae inoculated in the second instar. Error bars indicate SE. Values above bars indicate means. Columns headed by identical letters did not differ significantly (*t*-test, *p* > 0.05).

When applied as a diet surface contamination treatment, the presence of 1% glyphosate solution did not alter larval susceptibility to SfMNPV infection or virus replication in *S. frugiperda*. The prevalence of lethal polyhedrosis disease was 95–97% in the virus treatments, whereas none of the larvae died from virus infection in the glyphosate alone treatment or the control and non-specific mortality did not exceed 2%. The mean time to death varied from 100 to 108 h post-inoculation but did not differ significantly between the virus alone treatment and the virus + herbicide treatment (log-rank test *χ*^2^ = 1.108; d.f. = 1; *p* = 0.293) (Figure 3A). Mean larval weight at death was also similar between both treatments (*t* = 0.497; d.f. = 8; *p* = 0.633) (Figure 3B).

OB production/larva was nearly identical at 4.4 × 10^7^ OBs and 4.6 × 10^7^ OBs in the virus alone treatment and the virus + herbicide treatment, respectively (*t* = 0.152; d.f. = 8; *p* = 0.883) (Figure 3C).

### 3.4. Effects of Herbicide on Virus Acquisition on Treated Plants

**Figure 4:**
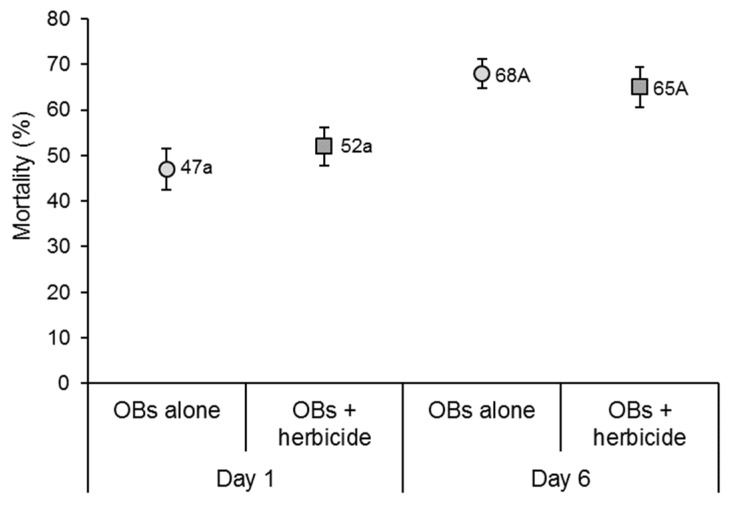
Mean percentage of virus-induced mortality of second instar larvae that consumed OB-treated maize plants to which glyphosate herbicide had been applied at 1 or 6 days previously in a greenhouse experiment. Error bars indicate SE. Mean values next to data points with identical upper or lower case letters did not differ significantly for comparisons within each time point (paired *t*-test, *p* > 0.05).

Of the insects that fed on Tween-treated or herbicide-treated maize plants without OBs, none died from virus infection and non-specific mortality was consistently less than 1% in both the Tween control and in the herbicide control. For the treatments involving OB application to maize plants, larvae that fed on plants treated with herbicide at 1 day previously experienced 52% lethal polyhedrosis disease, which was similar to that of larvae that fed on maize plants treated with OBs alone (paired *t* = 0.837; d.f. = 14; *p* = 0.417) (Figure 4). As mortality did not reach 50% in the OBs alone treatment, the median lethal time could not be compared for these treatments.

For the larvae that fed on plants treated with herbicide at 6 days previously, when the signs of glyphosate toxicity were becoming apparent, 65% developed lethal polyhedrovirus compared to 68% in the larvae that fed on plants treated with OBs alone (paired *t* = 0.734; d.f. = 14; *p* = 0.475) (Figure 4). The median lethal time of insects in the 6-day experiment was similar between the insects exposed to virus + herbicide-treated and plants treated with virus OBs alone (log-rank test *χ*^2^ = 0.121; d.f. = 1; *p* = 0.728).

### 3.5. Effects of Herbicide on Virus Persistence in Soil

The temperature of the experimental greenhouse varied from 8.9 to 37.3 °C with a mean (± SE) temperature of 20.4 ± 0.2 °C. Relative humidity varied from 20 to 100% with a mean of 76.5 ± 0.6%. The average (± SE) light readings ranged from 6 ± 1 lux at 06.00 to a maximum of 5993 ± 760 lux at 11.00 (Supplemental File S1). Light readings were unlikely to influence OB persistence in soil, as the plastic sample cups were opaque.

**Figure 5:**
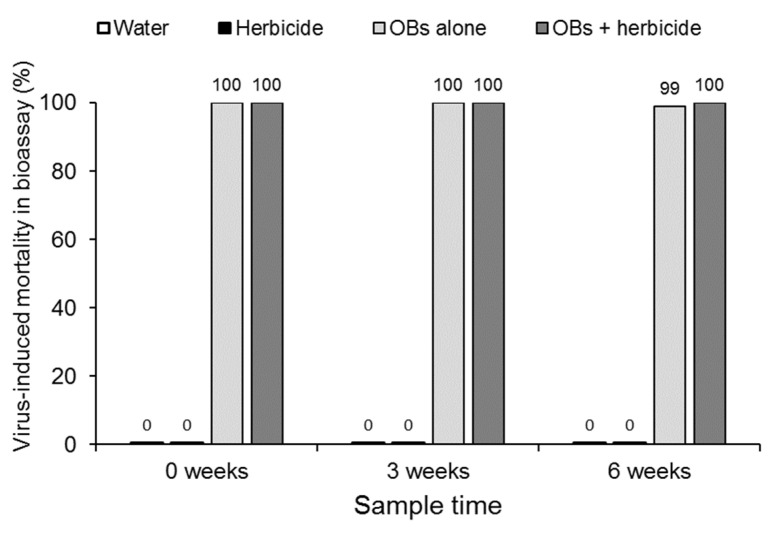
Prevalence of virus-induced mortality in soil bioassays of samples incubated in a greenhouse for 0, 3 or 6 weeks. Values above columns indicate mean percentages of four replicate samples from each treatment.

None of the larvae died from polyhedrosis disease in the water or glyphosate control treatments at any time point or in any replicate (Figure 5). In contrast, all the larvae succumbed to lethal polyhedrosis in the treatment involving OBs alone or OBs in mixtures with 2% glyphosate across all time points, with the exception of a single larva in the treatment involving soil + OBs alone in one sample taken at 6 weeks. These findings indicate that glyphosate herbicide had no effect on OB persistence in soil over the period of the experiment.

## 4. Discussion

In the present study, the effects of a glyphosate-based herbicide were determined on the growth and survival of *S. frugiperda* larvae, the pathogenicity of SfMNPV OBs, and on the acquisition of infection by larvae that consumed mixtures of herbicide and OBs on diet and treated plants and the effects on short-term persistence of SfMNPV OBs in soil.

We found no evidence for glyphosate herbicide effects on the survival or development of *S. frugiperda* larvae that fed on diet that had been surface contaminated with 1% (16 mM) herbicide solution. Application to the diet surface aimed to reflect the concentration of residues that larvae would be exposed to on treated foliage following a spray application of herbicide. In contrast, a recent study reported 50–80% mortality in glyphosate-treated eggs and larvae and a 30–50% reduction in progeny production in *S. frugiperda*, with similar results in the soybean pest *Chrysodeixis includens* [39]. However, these authors fully immersed eggs and larvae in herbicide solution for an undisclosed period at glyphosate concentrations of 0.2 and 4% (8.8 and 175 mM), resulting in the most pronounced adverse effects at the higher concentration. Although the mechanism of glyphosate toxicity in insects remains uncertain, molecular and physiological evidence for increased oxidative stress has been clearly observed in Lepidoptera, Diptera and Coleoptera following glyphosate treatment [40,41,42].

Studies on other orders of insects have reported reductions in mobility, food consumption, ovary development and adverse learning and memory effects across numerous studies, but often with inconsistent or conflicting findings due to differences in study design, toxicant dosage or methodology. Most of these studies have focused on honeybees, and to a lesser extent, drosophilid flies, beetles and aquatic insects [43].

To examine the direct effects of exposure to glyphosate herbicide on viral OBs, the OBs were incubated in herbicide solution (2%, 32 mM) for 24 h. Importantly, the herbicide was then removed by centrifugation, although traces may have remained in the liquid surrounding individual OBs. The OB suspension was then diluted 10^−1^–10^−5^-fold, further reducing any remaining glyphosate residue, and the OBs were then subjected to a standard droplet feeding bioassay. The control OBs were treated similarly. Given these steps, the exposure of bioassay insects to traces of glyphosate and any herbicide adjuvants will have been minimal. It is therefore unlikely that insect responses will have been affected by the presence of the herbicide. Following this procedure, we found no evidence for alterations in the pathogenicity of SfMNPV OBs, indicating that neither glyphosate nor the formulation adjuvants affected virus activity. The occlusion of ODVs within the polyhedrin matrix of the OB likely provided protection from potential harmful components in the product, and the slightly acidic pH of the solution (pH 5.78) was compatible with the integrity of OBs, which can be deactivated by strong acids and breakdown under alkaline conditions [30]. This is, to our understanding, the first study to examine the direct effects of a glyphosate herbicide on the infectivity of a virus.

We then tested the effect of insect susceptibility to infection in the presence of mixtures of SfMNPV OBs and herbicide residues (1% solution, 16 mM) on treated diet under laboratory conditions. Differences in the prevalence of lethal infection could have been due to changes in the midgut primary infection process, the immune response of the host, the feeding behavior in the presence of herbicide residues, or a range of additional effects. However, no such differences were detected. In a similar vein, although we suspected that larvae would show reduced virus acquisition on herbicide-treated maize due to reduced feeding on plants that showed signs of herbicide toxicity, no such difference was detected in the greenhouse experiment. Larvae were switched from feeding on maize leaves to semi-synthetic diet in the greenhouse experiment and may have experienced physiological stress while adapting to the change in diet. However, any debilitating effects of diet switching may have been offset by the increased protein content of the semi-synthetic diet compared to maize leaves, as diets with a higher protein/carbohydrate ratio tend to reduce virus-replication and increase insect survival [44,45].

We did not examine the midgut of *S. frugiperda* larvae, although gross pathological changes were observed in the midgut of *Bombyx mori* larvae that consumed glyphosate-treated mulberry leaves (3 g a.i./L, 13 mM) over a 48 h period [41]. Such changes in midgut integrity are unlikely to have played a role in our susceptibility experiment because studies on virus acquisition indicate that the majority of larvae become infected during the first few hours of feeding on OB-contaminated food [46]. After this time, larvae become less susceptible to infection as they develop both within each instar and from one instar to the next [47,48]. OBs on foliage also lose activity due to exposure to solar radiation and, in some cases, phylloplane chemistry [49], although this does not apply to our study performed on diet in the laboratory.

Reductions in immune function have also been reported in glyphosate-treated insects, including lepidopterans [42] and bees [50]. Glyphosate exposure promoted the replication of an iflavirus virus (*Iflaviridae*) in the honey bee [51], and increased the mortality of mosquito larvae exposed to a bacterial pathogen [52], but had no effect on a trypanosome gut parasite infection of the bumblebee, *Bombus terrestris* [53]. Marked changes in the gut microbiota have also been observed in bees and a coccinellid predator following exposure to glyphosate [19,54,55], including changes in the gut virome [56].

Two previous studies reported no significant changes in the susceptibility of lepidopteran larvae inoculated with their homologous nucleopolyhedroviruses, AgMNPV and ChinNPV, in mixtures with glyphosate-based herbicide [13,57]. Similarly, mixtures of SfMNPV OBs and atrazine or tembotrione herbicides did not result in changes in disease prevalence in *S. frugiperda* larvae, whereas mixtures of OBs and a soybean oil adjuvant with or without tembotrione had a significant deleterious effect for reasons that were unclear [17].

The study on OB persistence in glyphosate-contaminated soil was only of short duration but provided no evidence to suggest that glyphosate herbicide was detrimental to OB persistence in soil, even at the high concentration (2%) that we tested. The rate of degradation of glyphosate in soil is sensitive to temperature and soil moisture. In warm (20–30 °C), moist soil, glyphosate has a half-life of a few days, whereas the primary breakdown product, aminomethylphosphonic acid, has a half-life of several weeks or more under the same conditions [58,59,60]. This dissipation is mainly due to microbial activity [61]. For this reason, the soil persistence experiment was of relatively short duration, as soil OB populations are unlikely to be exposed to high concentrations of the herbicide for periods of more than a few days or weeks. In contrast, half-life estimates of nucleopolyhedrovirus OBs vary widely, from ~2 weeks to several months, depending on the virus and the type of ecosystem [62]. The retention of OB activity over the 6-week study, despite the fact that the soil was not previously sterilized and greenhouse temperatures reached up to 37 °C on some days (mean 20.4 °C), underlines the stability of OBs in the soil environment. Ultraviolet radiation was not a significant factor in the greenhouse experiment, as the soil samples were placed in opaque plastic pots in combination with the plastic structure of the greenhouse that greatly reduces ultraviolet levels inside the greenhouse.

The importance of soil as a reservoir for OBs has been little studied, despite the high insecticidal activity of isolates recovered from soil samples [63,64,65]. For example, between 5% and 60% of soil samples from maize fields in Mexico, Belize and Guatemala contained approximately 10^4^–10^5^ OBs/g of SfMNPV, which varied with soil type [31]. Similar soil OB concentrations were reported in other nucleopolyhedrovirus-host pathosystems [62]. In a recent study, analysis of over 30 viral meta-transcriptome samples from the soil of different crops revealed that baculovirus reads were the most prevalent reads in the soil virome and ranged from 35% of reads in rice soils to 48% of reads in maize soils in China [66]. In addition, long-term glyphosate use had no significant effect on the alpha or beta diversity of the soil virome in soils from soybean or maize fields [66], lending support to our findings on the low impact of this herbicide on soil OB persistence.

A laboratory study on the activity of AgMNPV revealed that OBs retained their activity significantly longer in soil or marsh water that had been autoclaved compared to untreated soil or water, suggesting that microbial activity was responsible for the observed effects [67]. Although many researchers have highlighted the diversity of the interactions that can arise following the introduction of glyphosate to agricultural ecosystems [68], detailed recent studies have concluded that the soil microbiome is not significantly affected by glyphosate [69,70], or where changes occur, these are mediated by microbial responses to glycine, an amino acid breakdown product of the herbicide [71]. The impact of other types of agrochemicals on the soil OB reservoir is almost entirely unknown despite considerable evidence of the adverse effects of many of these compounds on soil microorganisms [72].

As viral OBs experience harsher conditions in agricultural fields compared to laboratory and greenhouse environments, our findings should be verified across a range of crops and soil types with the aim of testing a range of climatic conditions (temperature, precipitation and incident sunlight), soil physico-chemical characteristics and biotic factors such as the plant phylloplane and soil microbial communities, all of which may influence the persistence of glyphosate residues and their interaction with viral OBs. An additional question concerns the possible role of formulation adjuvants such as surfactants, wetter-stickers, emulsifiers and others [73] that could also affect OB stability, given that OBs are sensitive to certain tensioactive agents [74]. As we only tested a single aqueous formulation (Takle 360 AS) for which the adjuvants are described as solubilizer and surfactant, the possible involvement of additional adjuvants in other types of herbicide formulations on the stability of glyphosate residues or their interaction with OBs remains to be clarified.

Given these findings, we conclude that the herbicide did not influence the development and survival of *S. frugiperda* larvae. Similarly, the physical stability, infectivity, acquisition of infection or soil persistence of SfMNPV OBs were not affected by exposure to the herbicide at the recommended application rates under laboratory and greenhouse conditions. Future studies should examine herbicide–OB interactions across a range of field conditions, soil types and different types of product formulation.

## Figures and Tables

**Table 1:** Mean (±SE) values related to larval survival, growth and development in insects that fed on glyphosate treated diet or an untreated control diet.

Variable	Control (Mean ± SE)	Herbicide (Mean ± SE)
Larval survival to pupa (%) ^1^	84.7 ± 0.05	88.9 ± 0.04
Time to pupation (d)	8.8 ± 0.1	8.5 ± 0.1
Weight of pupa (mg)	258.1 ± 3.4	253.3 ± 3.1
Duration of pupal stage (d)	8.9 ± 0.2	8.8 ± 0.1
Sex ratio (% male)	58.8 ± 12.7	50.0 ± 6.8

^1^ The SE values of binomially distributed means were calculated as √(p [1 − p]/t), where p = proportion of surviving insects and t = total number of larvae sampled.

## Data Availability

The original contributions presented in this study are included in the article/Supplementary Material. Further inquiries can be directed to the corresponding author.

## Supplementary Materials

The following supporting information can be downloaded at: https://www.mdpi.com/article/10.3390/insects17010073/s1, Table S1: Semi-synthetic diet for *Spodoptera frugiperda* larvae; Table S2: Analysis of the morphological and physico-chemical characteristics of the soil used in OB persistence experiment; File S1: Original data from the experiments performed in the present study.

## Abbreviations

The following abbreviations are used in this manuscript:

AgMNPV	Anticarsia gemmatalis multiple nucleopolyhedrovirus
ChinNPV	Chrysodeixis includens nucleopolyhedrovirus
GLM	generalized linear model
OB	virus occlusion body
ODV	occlusion-derived virion
SfMNPV	Spodoptera frugiperda multiple nucleopolyhedrovirus
