# Supplementary material for: Direct and Indirect Effects of a Glyphosate-Based Herbicide on Spodoptera frugiperda Multiple Nucleopolyhedrovirus (Baculoviridae) on Diet, Maize Plants and Soil
Source: Insects. 2026 Jan 8;17(1):73. doi: 10.3390/insects17010073 (PMC12842484; doi:10.3390/insects17010073)
Supplement: Supplementary file 1 [file insects-17-00073-s001.zip › Table S1.pdf]

Table S1. Semi-synthetic diet for *Spodoptera frugiperda* larvae

To prepare ~1 L of diet:

Ingredient	Quantity
Soybean flour	81 g
Wheatgerm	32 g
Brewer's yeast	25 g
Sucrose	13 g
Carrageenan	28 g
Potable water	1000 mL
*Potassium sorbate	1.0 g
*Methyl paraben	1.6 g
*Ascorbic acid	4.3 g
*Multivitamin mixture (Vitafort-A, Parfarm, Mexico City)	1.0 g
*Oxytetracycline chlorhydrate	130 mg
*25% acetic acid solution	12 mL
*15% choline chloride solution	7.3 mL
**10% formaldehyde solution	4.4 mL
***Mixture of salts	8.0 g

* Compounds are mixed in a 50 mL volume of water and added when the diet is at 70 °C and then cooled rapidly.

** Formaldehyde is not included in diet destined for bioassays.

*** Simplified mixture of salts comprising 21 g $CaCO_3$; 1.47 g $Fe_2(SO_4)_3$; 9 g $MgCl.nH_2O$; 12 g KCl; 31 g K_2HPO_4 (anhydrous); 10.5 g NaCl; 14.9 g $Ca_3(PO_4)_2$
