# Supplementary material for: Direct and Indirect Effects of a Glyphosate-Based Herbicide on Spodoptera frugiperda Multiple Nucleopolyhedrovirus (Baculoviridae) on Diet, Maize Plants and Soil
Source: Insects. 2026 Jan 8;17(1):73. doi: 10.3390/insects17010073 (PMC12842484; doi:10.3390/insects17010073)
Supplement: Supplementary file 1 [file insects-17-00073-s001.zip › Table S2.pdf]

Table S2. Analysis of the morphological and physico-chemical characteristics of the soil used in OB persistence experiment.

Morphological description

Depth	Horizon	Description
0-8 cm	A1	Light olive brown (2.5Y 5/4, dry) to dark brown (10YR3/3, moist), organic. Texture clayey. Granular structure fine with subangular boulders, moderately developed. Porosity fine interstitial tubular; adhesive and plastic. Resistance to breakage: firm. Large weathered stones, moderately abundant. Fine roots common, coarse roots common. Lower limit of horizon - clear and straight.

Textural characteristics

Horizon	True density (g/cm^3)	Apparent density (g/cm^3)	Total porosity (%)	Ksat (cm/h)	Clay (%)	Silt (%)	Sand (%)	Textural class
A1	2.5	0.7	66.0	-	69.6	21.3	9.1	Clay

Water retention characteristics

Horizon	Total moisture	Saturation moisture	Field capacity	Permanent wilting point	Residual mositure
A1	37.0	120.9	56.3	50.6	10.2

All values % vol./vol.

Cationic characteristics

Horizon	K	Na	Ca	Mg	Al	Cation exchange capacity	Soil brightness index
A1	2.66	2.55	7.53	4.45	0.05	24.48	17.19

Exchangeable cations in cmol/kg

Other characteristics

Horizon	Total saturated bases (%)	Effective cation exchange capacity (cmol/kg)	Organic material (%)	Carbon (%)	Nitrogen (%)	C/N	pH H_2O	pH KCl
A1	70.2	17.3	5.44	3.16	0.298	10.6	5.2	4.65

The soil was air dried and passed through a 1 mm sieve prior to use in experiments.

Data from Arreola-A.-Flores [36].
